# Supplementary material for: Two Different Isocitrate Dehydrogenases from Pseudomonas aeruginosa: Enzymology and Coenzyme-Evolutionary Implications
Source: Int J Mol Sci. 2023 Oct 7;24(19):14985. doi: 10.3390/ijms241914985 (PMC10574006; doi:10.3390/ijms241914985)
Supplement: Supplementary file 1 [file ijms-24-14985-s001.zip › ijms-2623978-supplementary.pdf]

Table S1. Primers for the construction ofwild-type and mutant *PET-28b (+)-PaIDH1.*

Primers	Sequences (5'- 3')
PaIDH1-F	GGAATTCCATATGGGATACCAAAAGATCCAGGTGC
PaIDH1-R	ATACCGCTCGAGTTACATCTTGGCAATCATCG
K346D-F	CCCACGGCACCGCGCCGGATTACGCCGGCCAGGACAAG
K346D-R	ATCCGGCGCGGTGCCGTGGGTCGCCTCG
K346D/Y347I-F	CCCACGGCACCGCGCCGGATATTGCCGGCCAGG
K346D/Y347I-R	AATATCCGGCGCGGTGCCGTGGGTCGCCTCG
K346D/Y347I/V353A-F	GCCGGCCAGGACAAGGCAAACCCGGGTTCG
K346D/Y347I/V353A-R	TGCCTTGTCCTGGCCGGCAATATCCGGCG
Y393K/R397S-F	CGCCGCCAAGACCGTGACCAAAGACTTCGAAAGCCTGATGGACGGCGCG
Y393K/R397S-R	CGCGCCGTCCATCAGGCTTTCGAAGTCTTTGGTCACGGTCTTGGCGGCG

Table S2. Primers for the construction ofwild-type and mutant *PET-28b (+)-PaIDH2.*

Primers	Sequences (5’- 3’)
PaIDH2-F	GGAATTCCATATGTCCATCCGCTCGAAGATCACC
PaIDH2-R	ATACCGCTCGAGTTAGGCCAGGCTGTCGATCG
H589L-F	CGGCTCGGCACCCAAGCTGGTCCAGCAAC
H589L-R	CAGCTTGGGTGCCGAGCCGCCGGCGCCGG
R600D-F	GGAAGAGAACTACCTGCTGTGGGACTCCC
R600D-R	CAGCAGGTAGTTCTCTTCCACCAGTTGCTGG
R649S-F	CAACAAGTCGCCGTCGAGCAAGGTCGGCGAC
R649S-R	GCTCGACGGCGACTTGTTGTTGTCCAGCAG
R600L-F	CTGGTGGAAGAGAACTACCTGCTGTGGGACTCCCTGGG
R600L-R	CAGCAGGTAGTTCTCTTCCACCAGTTGCTGG
R600I-F	CTGGTGGAAGAGAACTACCTGATTTGGGACTCCCTGGG
R600I-R	AATCAGGTAGTTCTCTTCCACCAGTTGCTGG
R649L-F	CAACAAGTCGCCGTCGCTGAAGGTCGGCGAC
R649L-R	CAGCGACGGCGACTTGTTGTTGTCCAGCAG
R649D-F	CAACAAGTCGCCGTCGGATAAGGTCGGCGAC
R649D-R	ATCCGACGGCGACTTGTTGTTGTCCAGCAG

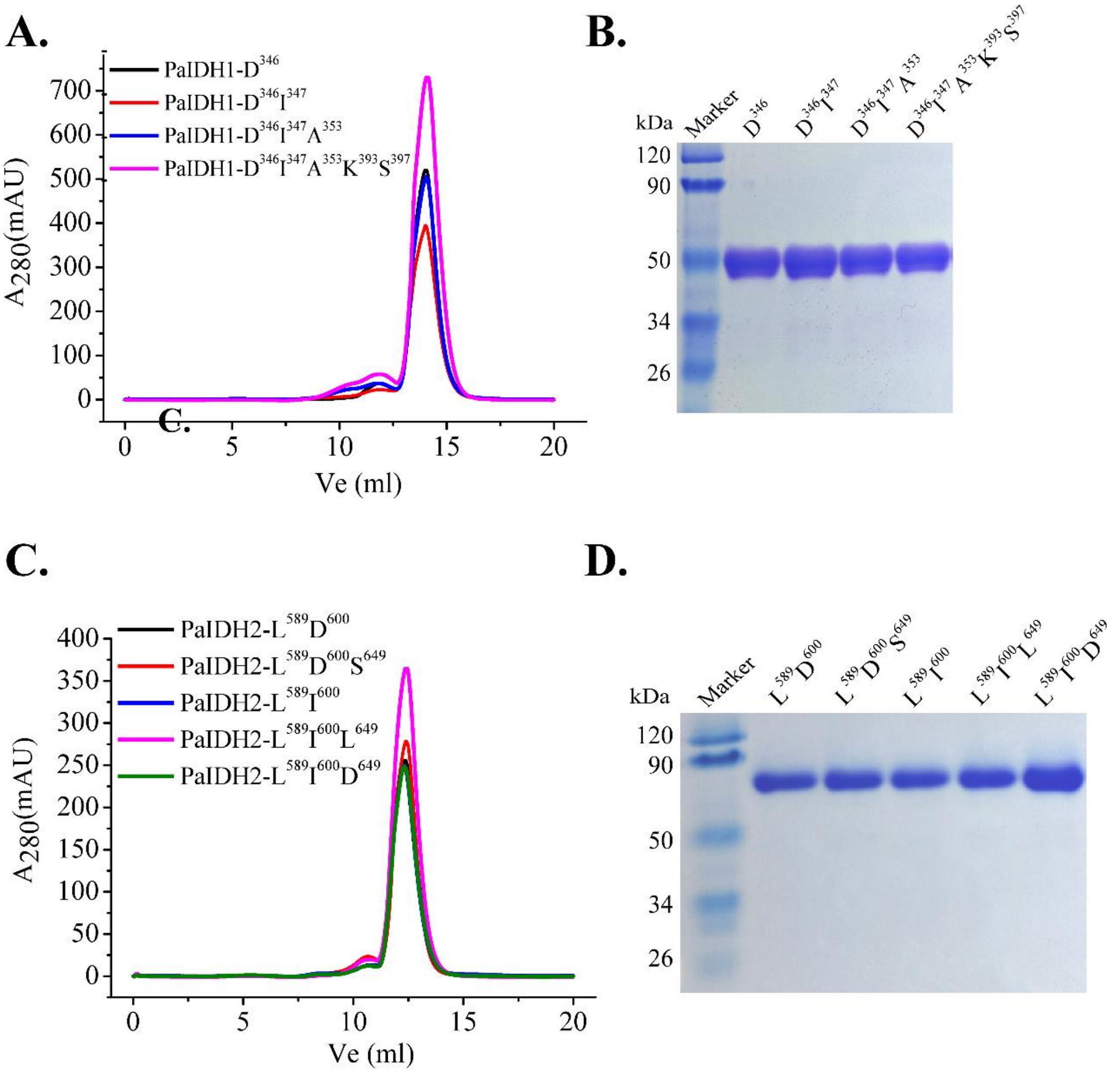

Figure S1. Determination of the molecular mass of PaIDH1 and PaIDH2 mutants. The flow rate was 0.5ml/min and the proteins in the fractions were monitored at 280 nm. (A) Result of gel filtration chromatography of PaIDH1 mutants. (B) SDS-PAGE of PaIDH1 mutants. (C) Result of gel filtration chromatography of PaIDH2 mutants. (D) SDS-PAGE of PaIDH2 mutants.
